# Mapping the Emergence of Synthetic Biology

**DOI:** 10.1371/journal.pone.0161522

**Published:** 2016-09-09

**Authors:** Benjamin Raimbault, Jean-Philippe Cointet, Pierre-Benoît Joly

**Affiliations:** INRA - LISIS, UPEM, Champs sur Marne, France; Imperial College London, UNITED KINGDOM

## Abstract

In this paper, we apply an original scientometric analyses to a corpus comprising synthetic biology (SynBio) publications in Thomson Reuters Web of Science to characterize the emergence of this new scientific field. Three results were drawn from this empirical investigation. First, despite the exponential growth of publications, the study of population level statistics (newcomers proportion, collaboration network structure) shows that SynBio has entered a stabilization process since 2010. Second, the mapping of textual and citational networks shows that SynBio is characterized by high heterogeneity and four different approaches: the central approach, where biobrick engineering is the most widespread; genome engineering; protocell creation; and metabolic engineering. We suggest that synthetic biology acts as an umbrella term allowing for the mobilization of resources, and also serves to relate scientific content and promises of applications. Third, we observed a strong intertwinement between epistemic and socio-economic dynamics. Measuring scientific production and impact and using structural analysis data, we identified a core set of mostly American scientists. Biographical analysis shows that these central and influential scientists act as “boundary spanners,” meaning that their importance to the field lies not only in their academic contributions, but also in their capacity to interact with other social spaces that are outside the academic sphere.

## Introduction

**Fig 1:**
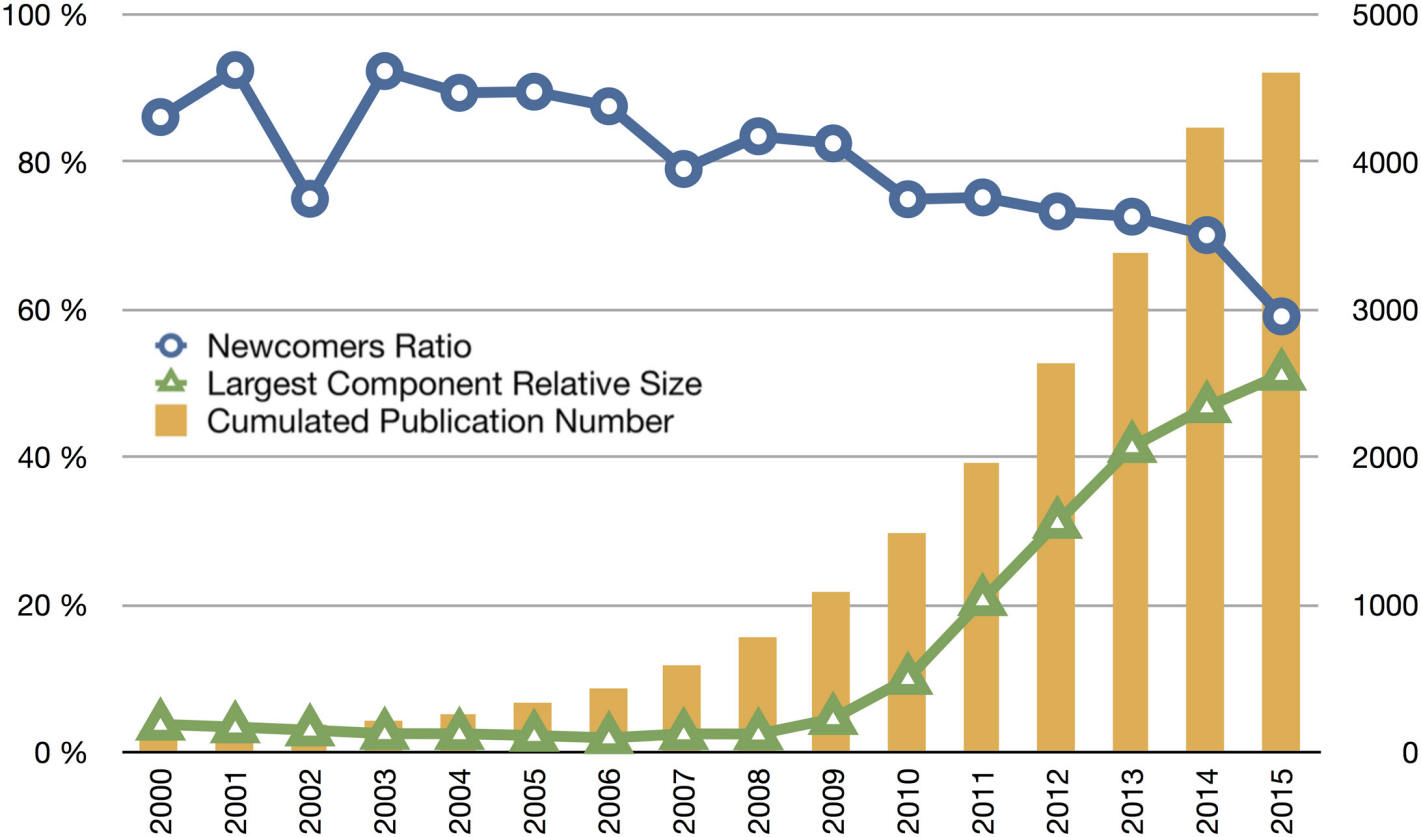
Global population statistics of the SynBio community over time. Cumulated number of publications (bar chart), newcomers’ ratio and largest component relative size. While the number of publication follows a typical exponential growth, the newcomers ratio, while very high, is decreasing with time, and the largest component relative size has been significantly growing since 2010 indicating a progressive structuration of the SynBio community.

**Fig 2:**
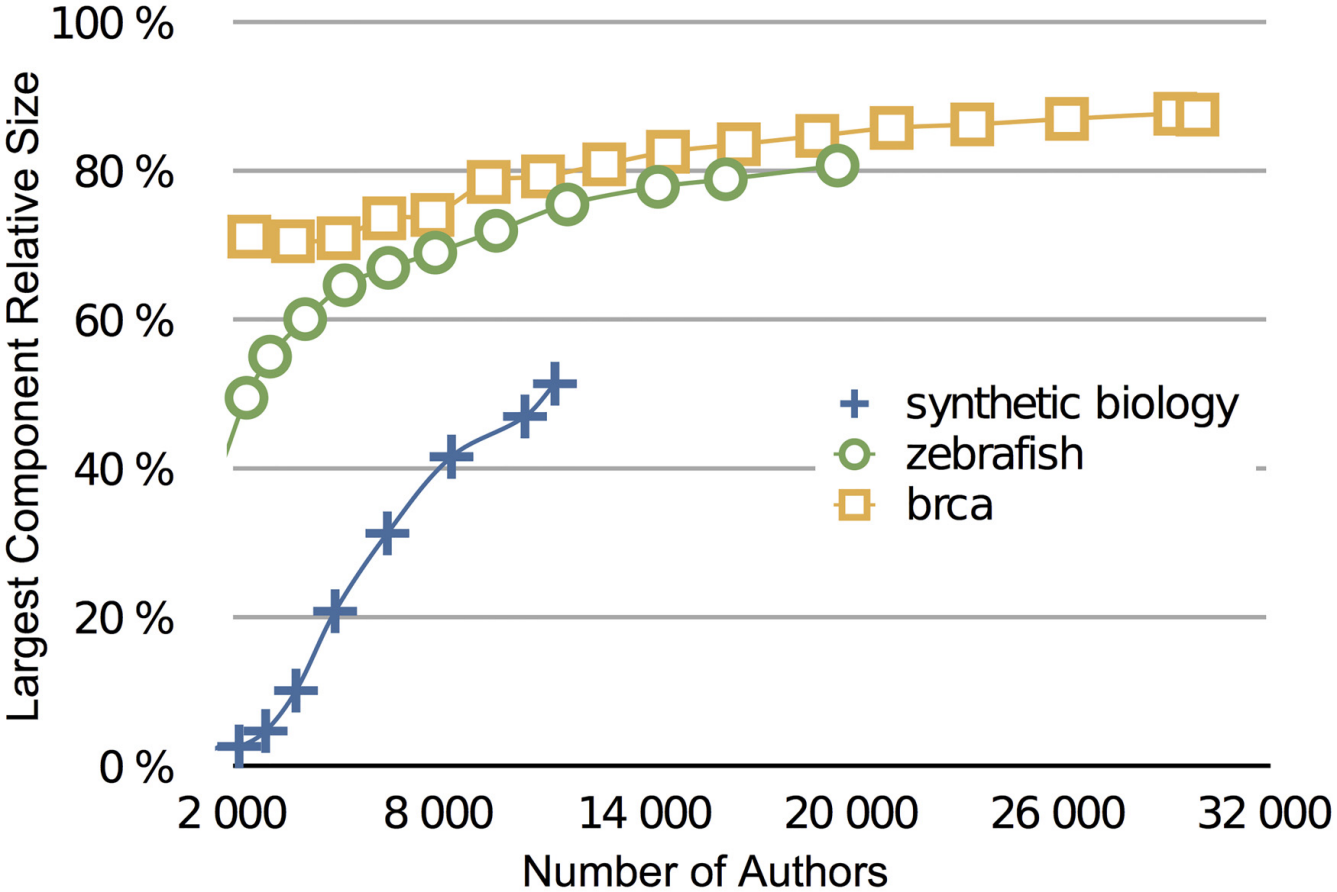
Phase diagram plotting the connected component relative size according to the cumulated number of authors at different years. Zebrafish and BRCA scientific communities were also plotted for the sake of comparison. SynBio (SB blue crosses) exhibits less “structuration” than those other fields for the same population size, but the gap is rapidly decreasing.

**Fig 3:**
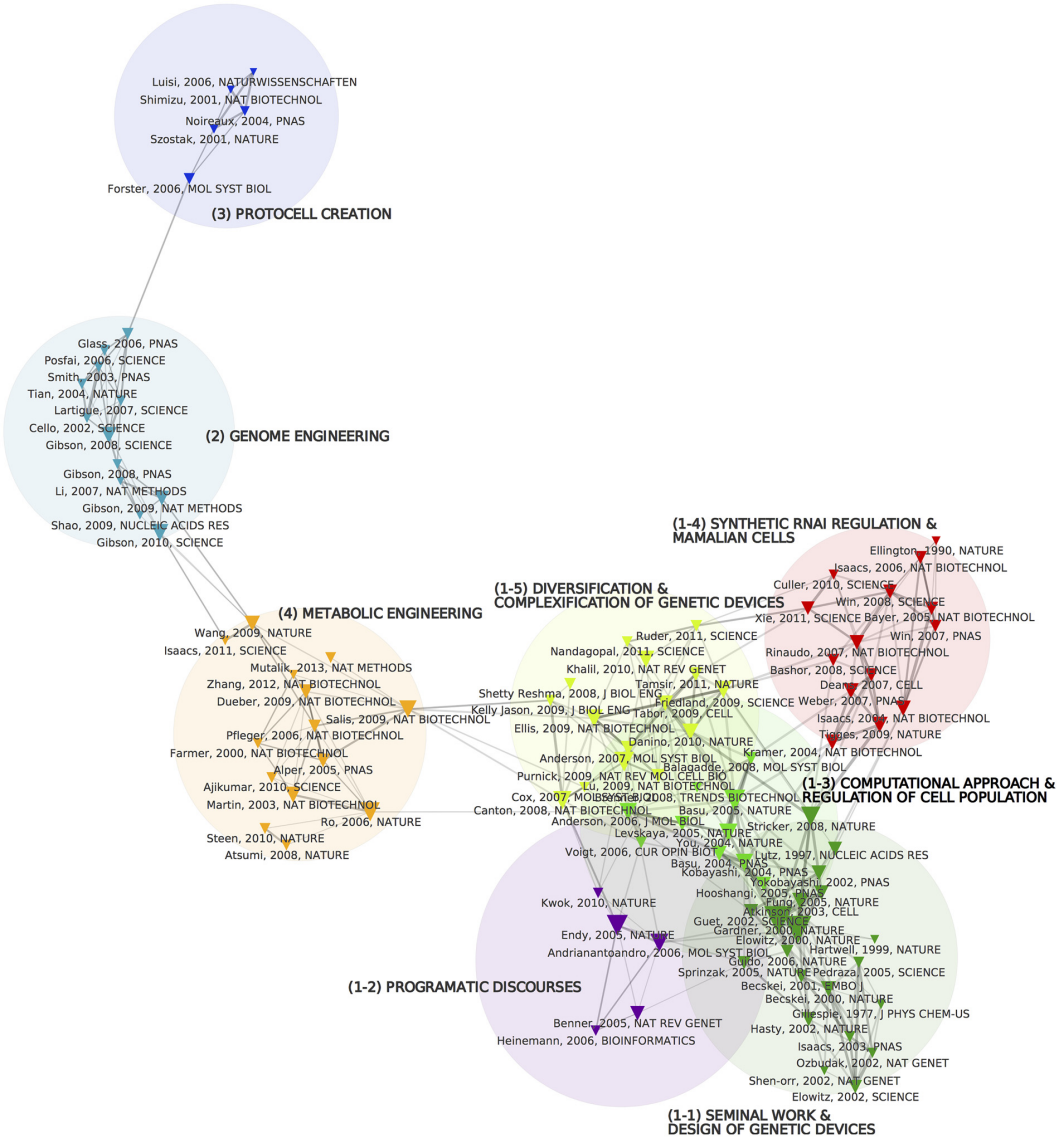
References co-citation map. The 100 most cited references are considered to build a co-citation network. Node sizes scale with the number of citations received by references. Node color depends on their cluster assignment. A colored circle is also drawn at the barycenter of each cluster which size is proportional to the number of related papers (that is the number of papers citing a significant number of references from this cluster). Edge widths are proportional to the strength of the link between references. Cluster labels (bold and upper-case) were manually added. During this labelling process, we gathered 5 very tightly connected clusters under the same family.

**Fig 4:**
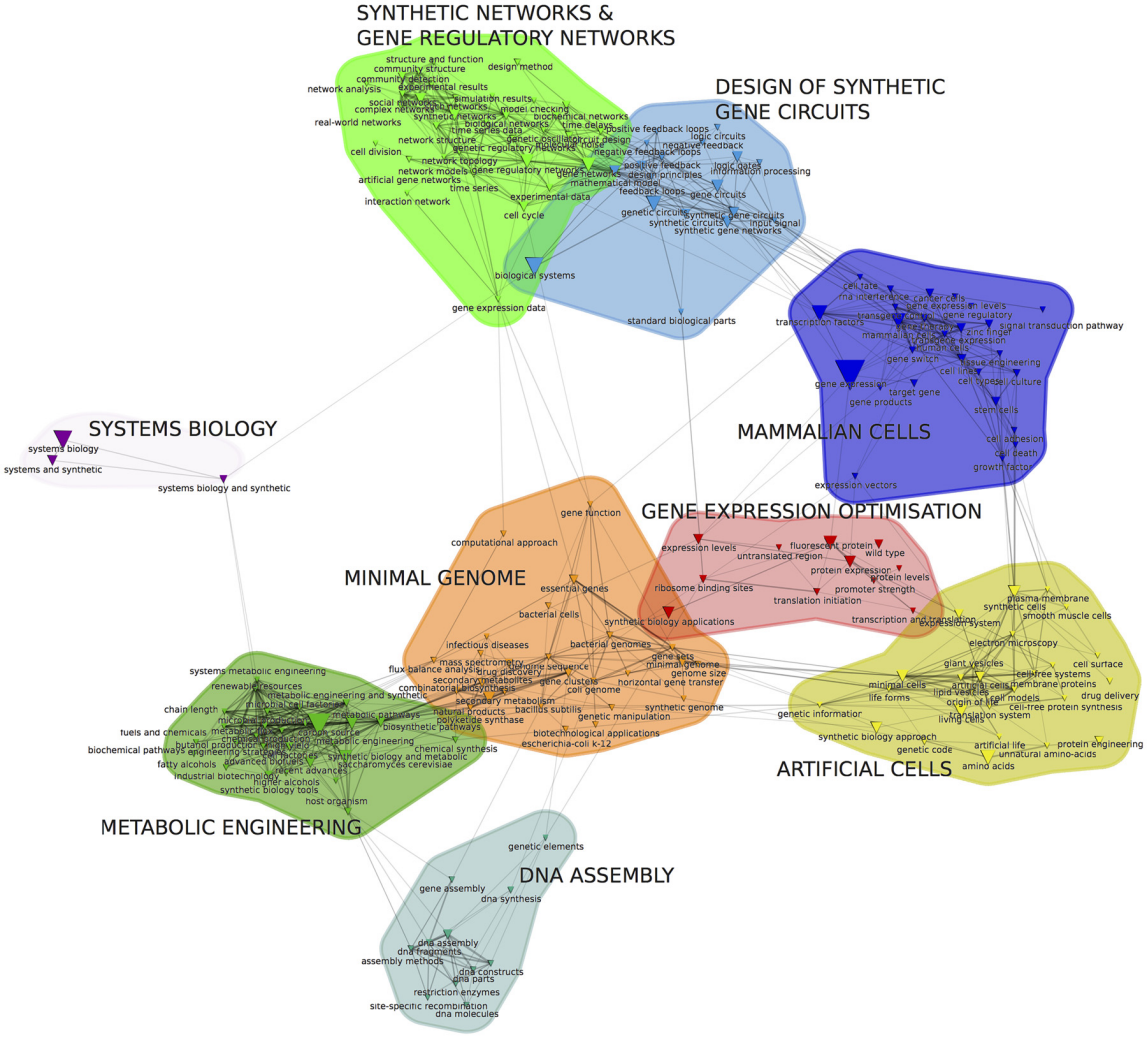
Semantic map showing the structure of 200 most pertinent terms extracted from titles, abstracts and keywords in the corpus. While some semantic clusters seem to be clearly resonant with co-citation clusters (Artificial cells/ (3) protocol creation), others are more instrumental or seem more conceptual (e.g. gene expression optimization, system biology).

**Fig 5:**
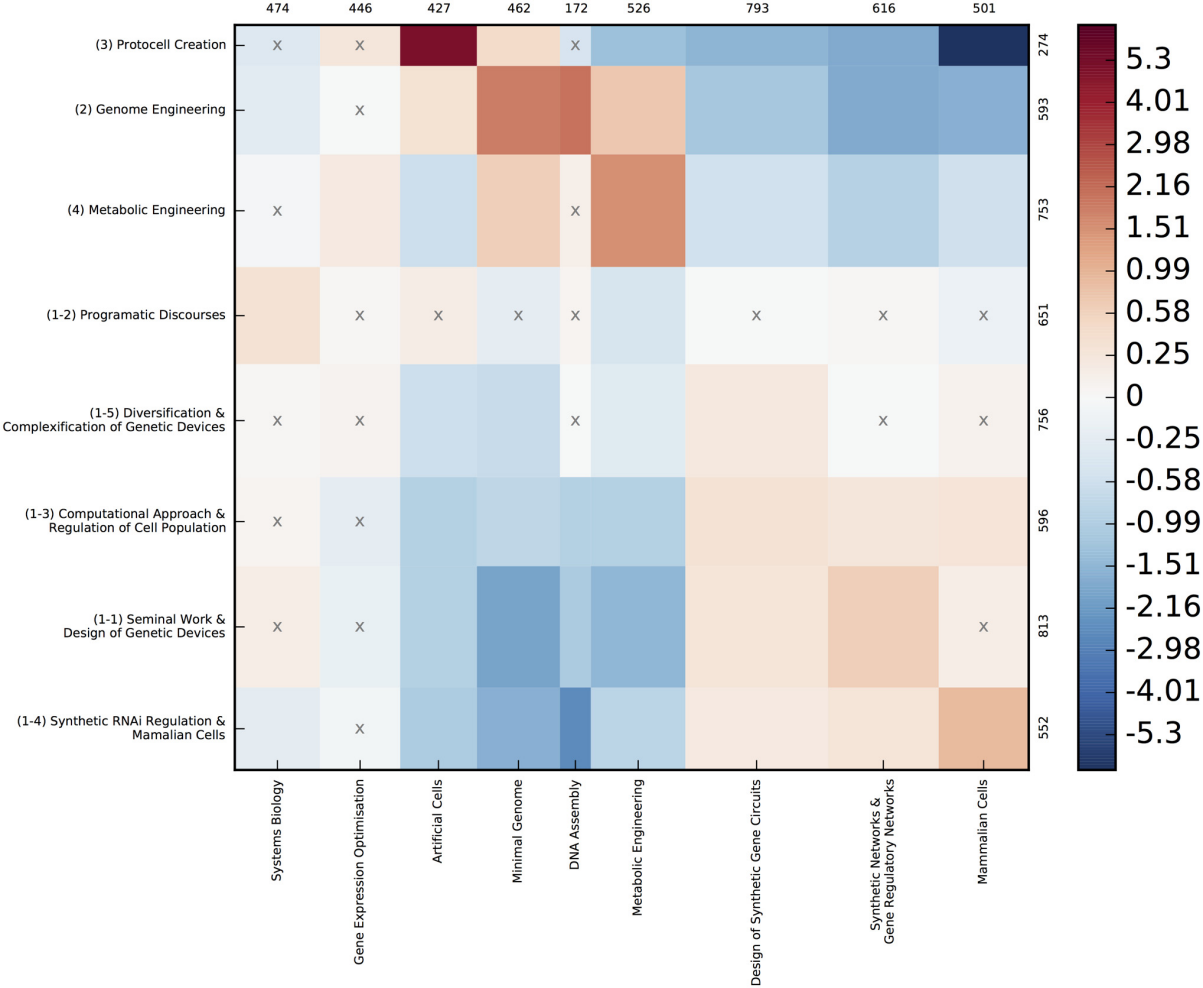
Contingency matrix between co-citation and semantic clusters. Each cell in the matrix shows how correlated/uncorrelated/anti-correlated a co-citation and semantic cluster are, meaning that the number of papers assigned to both clusters is higher/lower than the expected value we could expect from a random model. Red cells code for a positive deviation, while blue cells indicate a negative deviation. The intensity of colors shows the intensity of the deviation. For instance the deepest red cell shows that there are more than five times more papers assigned to both “(3) Protocell creation” and “Artificial Cells” than expected if co-citation and semantic cluster distributions were independent, conversely “Protocell creation” is anti-correlated with “Mammalian cells” (Five times fewer papers than expected. Crosses indicate cells where the deviation from the null model is not statistically significant).

**Fig 6:**
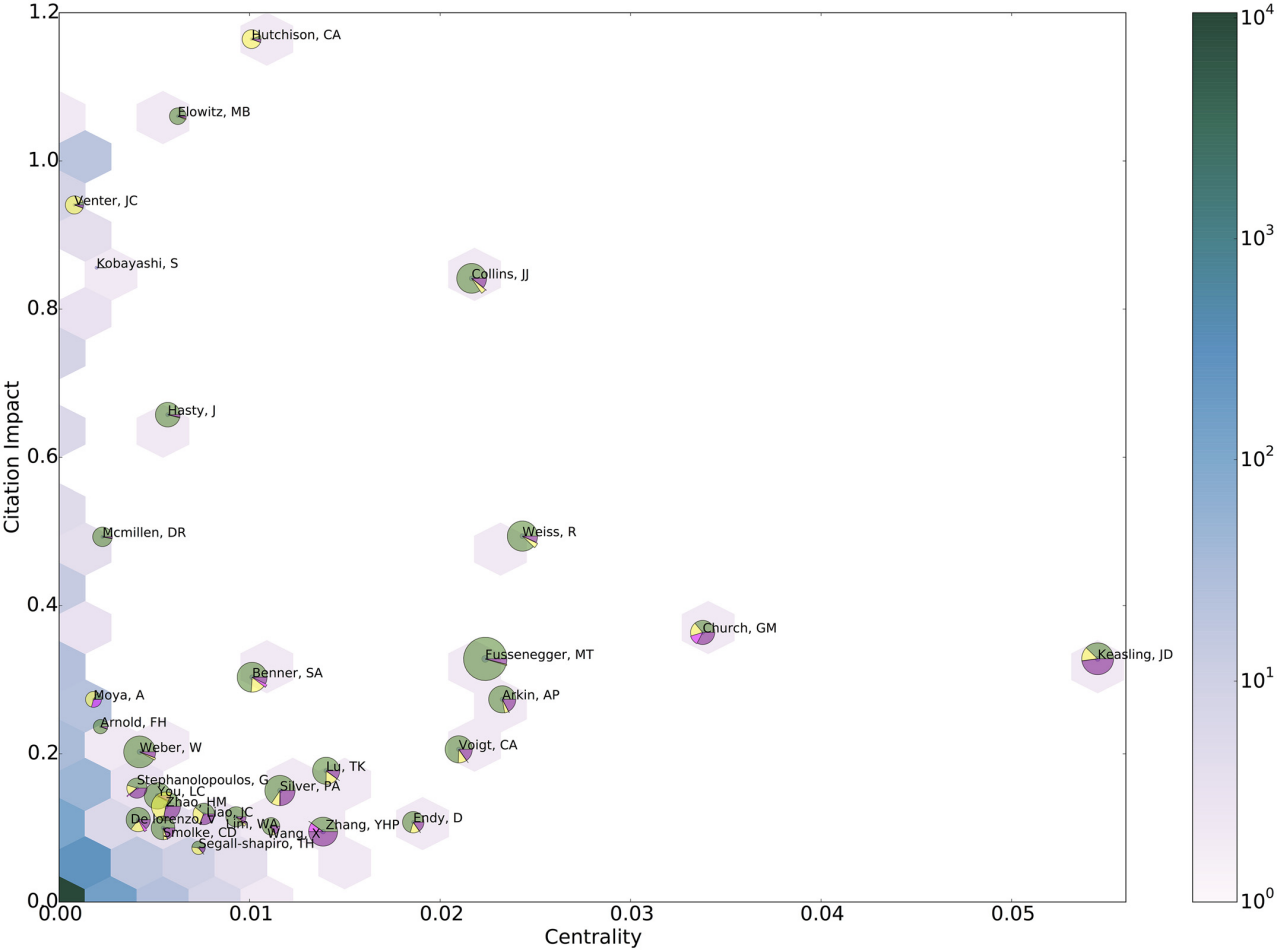
Position analysis of the main actors of SynBio community according to their centrality and impact. The joint distribution of nodes betwenness centrality and cumulated Impact is represented through the hexagonal heatmap showing how concentrated the population is: more than 10000 scientists (over 11321) actually have both a very low impact and centrality. Conversely, the 30 most important scientist (according to the product of impact and centrality) are plotted with a pie diagram showing their paper distribution among the 4 different approaches (green for Biobricks engineering, yellow for genome engineering, purple for metabolic engineering and pink for protocell creation). The epistemic landscape is strongly defined by members of this core-set: they have indeed co-authored 77 over the 100 most cited articles by the corpus.

Although the first use of the term synthetic biology in the scientific literature dates back to the early 20th century, contemporary synthetic biology started to bloom around the turn of the new millennium and has been presented as “novel, perhaps revolutionary, and cool” [1]. Synthetic biology, like most emerging fields, can be defined in numerous ways since the definitions come from members belonging to a self-selected community in the making. The EC Opinion on Synthetic Biology [2] identified 35 published definitions and proposed the following: “SynBio is the application of science, technology and engineering to facilitate and accelerate the design, manufacture and/or modification of genetic materials in living organisms”. Synthetic biologists suggest that compared to modern biotechnology (e.g., genetic engineering, genomics, high throughput biology, etc.), the epistemic novelty of SynBio lies in the systematic use of engineering approaches to intentionally design artificial organisms. However, the meanings of engineering are actually very diverse. As a result, biologists and social scientists working on SynBio acknowledge this heterogeneity, and they often distinguish between three main approaches: DNA-based construction (another naming convention for a “biobrick engineering approach”), genome driven cell engineering, and protocell creation [3]. SynBio can be differentiated according to two visions of engineering namely a modular vision inspired from informatics and a vision based on the analogy with synthetic chemistry [4]. In this paper, we claim that such heterogeneity plays a constitutive role in the emergence of SynBio (and more generally of any emerging field) and one of our core objectives is to characterize this heterogeneity in a robust way. An emerging scientific field has to be built on really new groundbreaking ideas, but these novel ideas have to be credible enough to attract resources [5]. Hence, an emerging field is made up of a mix of novel high-risk research lines and pre-existing tracks that are re-labeled. The constraint of credibility applies firstly among scientific communities, but also touches upon other audiences, for example: public authorities dealing with science policy, big pharma, venture capital, the general public, etc. Hence, a complementary reason that reinforces heterogeneity is related to the need to formulate promises to address large societal challenges. As such, SynBio is then better defined as an umbrella term (like nanotechnology or sustainability research) that gathers a set of activities that ranges from the basic sciences to innovative technology [6], rather than as a new scientific paradigm [7]. If heterogeneity is a constitutive dimension of the emergence process, then it is also a potential hurdle for the stabilization of the field. This leads to a tension between being open enough to new participants and conducting boundary work [8]. In this paper, we make use of original scientometric analyses to study this constitutive role of heterogeneity. The methods are outlined in the first section. The results are then presented as follows. We first analyse the dynamics of the population of scientists. We show how since 2010, SynBio began stabilizing as an autonomous scientific field, while maintaining a high level of openness to new ideas and participants (Figs 1 and 2). Second, the core of the paper is devoted to a description of the epistemic heterogeneity of the field. The use of original and rigorous approaches based on co-citation and lexical networks allows for the identification of a set of epistemic clusters and the characterization of their relative positions (Figs 3, 4 and 5). Our third analysis draws on a scientometric approach to identify a “core set” of highly influential scientists (Fig 6 and Table 1). Using biographical data, we analyse how they contribute to build an environment that may foster the development of SynBio.

**Table 1:** Repartition of the core-set members according to the epistemic approaches (Appro.) (BE = Biological Engineering, ME = Metabolic Engineering, GE = Genome Engineering and PC = Protocell Creation) of SynBio identified previously and to their involvement in non-academic activities according to three indicators: Business Development (Bus.), Core-Institutions (Inst.) and Governance (Gov.). The presence of a * indicates the participation of the author to the Synthetic Biology Engineering Research Center (SynBERC).

PI	Institutions	Appro.	Bus.	Inst.	Gov.
CA Voigt*	MIT, USA	BE	3	4	1
P Silver*	Harvard University, USA	BE	2	4	2
D Endy*	Stanford, USA	BE	3	3	6
J Keasling*	Berkeley National Lab, USA	BE ME	3	3	2
G Church*	Wyss Institute, USA	BE ME PE	3	3	2
J Collins*	MIT/Wyss Institute, USA	BE	3	2	1
R Weiss*	MIT, USA	BE	2	2	2
A Arkin*	Berkeley, USA	BE	2	2	0
W Lim*	UCSF, USA	BE	1	2	1
M Fussenegger	ETH Zuich, SW	BE	3	2	0
F Arnold	Caltech, USA	BE	3	2	0
C Smolke	Stanford, USA	BE	3	2	1
J Hasty	UCSD, USA	BE	2	1	0
T Lu	MIT, USA	BE	3	1	0
X Wang	Arizona State Univ., USA	BE	1	1	0
D Mcmillen	Toronto, CA	BE	1	1	0
W Weber	BIOSS (Freiburg), SW	BE	3	1	0
L You	Duke University, USA	BE	0	1	0
G Stephanopoulos	MIT, USA	BE	3	1	0
T Segall-Shapiro	MIT, USA	BE GE	1	0	0
J Liao	UCLA, USA	BE GE ME	3	0	0
M Elowitz	Caltech, USA	BE	2	0	1
S Benner	FAME, USA	BE	3	0	0
V De Lorenzo	CNB Madrid, SP	BE	3	0	2
Y Zhang	Virginia Tech, USA	BE ME	2	0	0
C Hutchison	JCVI, USA	GE	2	0	0
C Venter’	JCVI, USA	GE	3	0	1
H Zhao	Illinois University, USA	GE ME	2	0	1
C Moya	University of Valencia, SP	PC GE	0	0	0

### Methods

We claim that quantitative analysis of scientific publications is a privileged way to unveil the structuration dynamics of an emerging scientific field. However, traditional bibliometric tools have two caveats that need to be overcome. First, most of the time they are built on already stabilized categories such as with keywords defined by librarians. Synthetic biology obviously cannot be accurately described with such descriptors, as its categories are not yet stabilized. Second, traditional methods usually fail to capture the dynamic patterns underlying knowledge circulation between publications.

Contrary to previous scientometric studies [9], MIT website (http://web.mit.edu/demoscience/SynthBio/players.html), this paper focuses on the process of structuration and highlights the heterogeneous composition of the field. It is the first time that the dynamic process regarding the emergence of SynBio has been thoroughly analyzed with a systematic use of scientometric data. This use of innovative modeling and visualization capacities allows for the mapping of original textual entities pertaining to a publication’s raw content (analyzed with natural language processing tools) and citation cascades. This section provides a brief account of the technical choices made for defining our corpus, and introduces our mapping strategy.

#### Corpus delineation

Defining a pertinent corpus of publications was the first major challenge that had to be addressed. We built an initial core corpus comprising articles extracted from the scientific publication database Thomson Reuters Web of Science. The articles matched the simple query condition: “Topic = synthetic biology”. This first core corpus thus consisted of every article available on the platform that contained the term “synthetic biology” in the textual content of the bibliographic record. As we assume that authors working in the domain may not systematically use the expression “synthetic biology”, we decided to perform a lexical expansion [10] allowing us to identify 11 supplementary terms specific to the field (see S1 File for detailed procedures). Additionally, this list was independently validated by four experts from the domain. Our final query was thus: TS=“synthetic biology” OR “synthetic gene network” OR “Standard Biological Parts” OR “artificial genetic system” OR “synthetic genom*” OR “synthetic gene circuits” OR “minimal cells” OR “synthetic circuits” OR “synthetic networks” OR “synthetic cells” OR “minimal genome” OR “artificial gene networks”. Our final corpus was comprised of 4,605 publications. The corpus were built in July 2015. The query was applied to the field “TS” of the ISI, which allows one to search for any article containing one of the 12 terms under either title, abstract or keywords of the article. We also added wildcards when necessary to accommodate possible simple variations (genomic/genome). It is worth noting that our corpus is necessarily partly noisy. Although we may have tried alternative corpus delineation strategies (for instance [11]), our main claim is that the small proportion of articles which are not really Synthetic Biology papers have a negligible impact on the overall characterization of the field we provide (see S1 File for a quantitative evaluation of this effect).

#### Network analysis

The epistemic and cognitive landscapes of the SynBio community were mapped through an analysis of two networks: (a) co-citation and (b) lexical networks. An analysis of both networks and comparison allowed us to identify the diffusion of scientific credit, readily visible through the co-citation map, and the structuration of cognitive areas prominent in synthetic biology through the lexical (also called co-word) map. Technically, both the lexical and co-citation maps were produced by following essentially the same strategy:

1. We first selected the most frequent nodes (the top 100 most frequently cited references or the top 200 most frequent terms). Natural language processing tools were first applied to titles, abstracts and keywords of articles to extract the most pertinent multi-terms in the corpus (see S1 File).
2. lexical or co-citation network was constructed as follows. Two nodes (references or terms) were connected when they frequently appeared together in the same article (citations or in a terms list). A cooccurrence matrix was built, from which a proximity measure between each pair of nodes was computed (a classical cosine distance was applied for co-citation networks, and we used a slightly more elaborate measure than that used in information retrieval to build the lexical network) [12], [13].
3. The network structure was then analyzed with a community detection algorithm (also called a clustering algorithm). We utilized the Louvain algorithm to automatically reveal this high-level structure [12]. Finally, a dedicated spacialization algorithm enabled the arrangement of nodes in a representation optimizing the position of nodes such that their “geometric neighborhood” mimicked their “structural neighborhood” while coloring the automatically detected clusters.

Citation analysis is a traditional scientometric technique [14] that makes it possible to visualize clusters of related citations as the constituent sub-domains of a given research field. In our case we used the joint appearance of two cited references in the same reference list as the structuring principle of the co-citation network (as opposed to inter-citation analysis connecting citing and cited references). The rationale underlying co-citation analysis is that the structure of the co-citation network (detected through an automatic community detection algorithm) indexes the epistemic foundations of the research community, which consists of groups of seminal works shared by the various sub-communities (Fig 3). The co-citation map is also informative when it comes to understanding the relations between different epistemic groups. In line with co-word analysis, we claim that the vocabulary used in an article title and abstract is a satisfying proxy for describing its cognitive positioning. Lexical analysis reveals shared perspectives, discourses, metaphors or even technical tools used in the field. Natural Language Processing (NLP) provides a direct way for accurately describing ideas, concepts and instruments that researchers are using and combining in emerging fields. Terms list identified through NLP tools are extracted from the final corpus. They must be distinguished from the search terms used for extending the query which have been selected after a 3 steps process detailed in the S1 File. Lexical analysis allows us to: (1) identify the vocabulary of the field (where discursive elements, achievements and techniques are frequently being used in the publications); (2) detect the different lexical fields emerging from the analysis of the lexical network structure of synthetic biology; and (3) analyze how this new map overlaps or not with the epistemic landscape drawn from co-citation analysis. This comparison is made possible thru a contingency matrix (Fig 5, Methods section), which allows correlation of co-citation and lexical clusters (Figs 3 and 4). Each article is first assigned the lexical and co-citation clusters they are the closest to according to their vocabulary and reference list. We then compute the contingency matrix enumerating for any pair (c,s) (co-citation cluster c and lexical cluster s) for the actual number of papers which were assigned to these clusters. On the different maps, each node (either a citation or a term) is represented by a triangle whose surface is proportional to its frequency. The width of links connecting two nodes is proportional to their proximity measures strength. In order to provide an interpretation of these maps (especially the co-citation map), we read the abstracts and analyzed the biographies of the main authors. The clusters were then labeled on the basis of this interpretation (and shown in bold capital letters). These innovative mapping approaches have also been described in detail in [15]. Most of the maps presented here were obtained using the CorText Platform (http://www.cortext.net) developed by one of the authors. The contingency matrix (Fig 5) illustrates how correlated (and conversely anti-correlated) the distribution of articles over co-citation and lexical clusters are (see S1 File).

## Results and Discussion

### Corpus basic statistics

As of July 2015 a total of 4,605 publications were listed in Web of Science for our enriched query. The characteristics of the corpus are similar to those described by the previous quantitative analysis of Oldham et al. [9]. However, given the annual growth rate (about +34% since 2000), the number of publications in our corpus is almost twice the size of the corpus analyzed in the Oldham et al. paper. SynBio papers are published in journals belonging to a wide range of disciplines including: biochemistry and molecular biology (21%), biotechnology and applied microbiology (13%), science and technology (9%) and chemistry (6%). The high frequency of computer science, mathematical & computational biology, and engineering (14% of the “disciplines of origin”) confirms the importance of engineering in this field. Even if they enforce our argument on epistemic heterogeneity, those percentage have to be nuanced and considered only as crude indicators directly based on a Web of Science categorization [16]. Globally, the United States leads the production of scientific publications, representing 45, 6% of the production, followed by Europe with 41, 7% and finally Asia with 17,5%. The geographical concentration of research in SynBio is even more striking when considering the leading institutions. Among the 10 institutions that appear most frequently, eight are American universities. In Europe, one should mention the leading position of the United Kingdom (11,6% of publications and four universities in the top 20)

### Boundaries work: between permeability and structuration

The 4,605 scientific papers were authored by 11,348 scientists. In 2014 (last year for which the dataset can be considered as complete) there were a total of 3,044 unique authors, 2,133 of which are newcomers (they have never published before in the field). The proportion of newcomers in 2014 was very high (70%), but was still much lower than in 2004 (89%). This proportion has indeed slowly been decreasing since then (blue curve in Fig 1). Note that although the ratio of newcomers is decreasing, the number of new entrants is still consistently increasing every year. Data for the first half of 2015 even shows an acceleration of this decrease with a population of authors composed of only 59% of newcomers. The rapid decrease of the ratio of newcomers illustrates the growing structuration of the field. Moreover, the current proportion of scientists who publish only once is not significantly different from other scientific fields. Diana Crane [17] reports that in any scientific specialty ‘transients’ publishing only once represent 60% of the population. This high proportion of transients is partly due to the proportion of doctoral students and post docs, which is especially high in life sciences.

Another characteristic of a scientific community lies in its connectivity. A possible indicator is the relative size of the ‘largest component’, defined as the largest subset of researchers connected through a direct path in the collaboration network [18]. This indicator is a good proxy of the cohesion of the community. The green curve in Fig 1 shows that the relative size of the largest component has grown steadily since 2008 to reach up to 51% in 2015 (Fig 1). If we compare the dynamics of this largest component’s relative size to other communities of reference: BRCA and zebrafish (Fig 2), we can draw two interesting conclusions [19]. First, even when controlling for the number of authors—and the observation still holds when plotting the principal component relative size with the cumulated number of publications—SynBio’s largest component seems significantly smaller. We can infer that the heterogeneity of the field tends to create numerous small sub-groups that do not collaborate with each other. But despite this discrepancy, we also observe that the largest component relative size is rapidly increasing and is getting closer and closer to “normal proportions” of around 80% as measured in other life science fields. In line with the analysis by Bettencourt and Kaur [18] of the densification of a collaboration network in sustainability science, both characteristics (proportion of new entrants and size of the largest component) show that SynBio, although still attracting numerous newcomers, is maturing.

### A constitutive heterogeneity of core approaches

In the 1969 post-face to ‘The Structure of Scientific Revolutions’ Thomas Kuhn highlighted the importance of the study of scientific communities to understand the dynamics of science: ‘If this book were being rewritten, it would (…) open with a discussion of the community structure of science’ [20]. It is crucial to study the way groups of scientists emerge and develop shared visions of their research topic, and eventually create what Kuhn coined as a paradigm. But before turning to individual scientists as such, we first propose a systematic characterization of the SynBio community as a whole. We showed the progressive stabilization of SynBio around a growingly cohesive community of scientists. We then explore its epistemic and cognitive inner structure using co-citation and semantic maps (see Method section). A first key characteristic of the field is its high level of autonomy in the definitions of approaches and core concepts. Citation analysis shows that around half (48) of the top 100 papers cited by the papers of the corpus of SynBio actually belong to the corpus. Note that cited references may include publications that do not belong to the original corpus, thus referring to areas of knowledge that are not specific to the field. Although it is an emerging field, the scientific basis of SynBio are mainly within the field.

The co-citation analysis allows us to identify four different groups of cited references as plotted in Fig 3. One of these is actually composed of five tightly connected clusters (bottom right of Fig 3). The semantic map exhibits a different set of clusters (Fig 4) that are however highly correlated to the clusters of Fig 3 as appears in the contingency matrix (Fig 5). On this matrix, we also observe that the five tightly connected sub-clusters share the same vocabulary or at least are investigating very different topics than the three other clusters. This multi-level structure is confirmed by applying a Louvain community detection algorithm on the “high level network”, which groups together these five clusters. Hence, the combined use of co-citation and lexical analyses allowed us to identify four main approaches in the SynBio field. We coined them ‘approaches’ following Maureen O’Malley et al., although our quantitative analysis shows a somewhat different and richer structure. The four approaches are the following (Fig 3): biobrick engineering (Clusters 1-x), genome engineering (Cluster 2), protocell creation (Cluster 3) and metabolic engineering (Cluster 4). In the next paragraph, we will describe the structure and dynamics of each approach.

At the macro-level, our analysis reveals a highly asymmetric structure. “Biobrick engineering” approach (clusters 1-x) is much more highly cited and central than the genome engineering approach and “metabolic engineering” while “protocell creation” is peripheral. Biobrick engineering’ explicitly uses an electronic metaphor, introduces a set of new concepts such as gene circuits, genetic switches, biobricks, chassis, etc., and refers to modeling. Based on this and following Hilgartner [21], we describe the biobrick approach as the most visionary approach meaning that its vision for SynBio (programmatic discourses and direction for the field) affects interpretation of biology (example:modularity) or scientific practices (example standardization). One of its sub-clusters (1.2) is dedicated to ‘programmatic discourses’. This approach is foundational for SynBio and very specific to this new field. ‘Genome engineering’ (2) and ‘Metabolic engineering’ (4) are complementary and closely linked in the lexical space. These approaches (genome engineering and metabolic engineering), although related to SynBio, also belong to established scientific fields. Some of the research in these fields are being relabeled as SynBio (a classic phenomenon in the emergence phase of new scientific fields). Whereas the first approach provides the vanguard visions of the field, ‘metabolic engineering’ (and to a lesser extent ‘genomic engineering’) provide credibility for claims about applications. Significantly, the example of biosynthesis of artemisin is frequently used as a success story showing the potential of SynBio, although this was obtained with classical approaches of metabolic engineering. The later approach (Protocells—3) is devoted to the understanding of the origin of life through artificial reconstruction of living entities. This approach is not tightly linked with the others and it has weakened in the last 10 years.

#### Biobrick engineering approach

The biobrick engineering approach includes five sub-clusters (Figs 3 and 4). Cluster 1-2 (Programmatic Discourses) gathers programmatic papers that present the vanguard vision and the potential future of SynBio [22], [23] and [24]. These three articles were written shortly after 2004, which is considered as a milestone for institutionalization of the field [1]. Cluster 1-1 (Seminal works/design of genetic devices) includes scientific papers about foundational achievements that support this engineering vision [25, 26]. Two further clusters are built around some of the key components of the approach: gene circuits (1-3 computational approaches) with articles by Arnold (4), Weiss (3), Voigt (3) and You (3). and synthetic corpus, thus referring to areas of knowledge that are not specific to the field.

RNA to control gene expression in mammalian cells (1-4 RNAi/Mammalian cells) with articles by Smolke (4), Fussenegger (3), Collins (3) and Weiss (2). The last cluster includes papers that integrate different novel approaches for designing original applications (1-5 Diversification & complexification of genetic devices) We find again a relative homogeneity with four authors representing the majority of the articles Collins (5 articles), Voigt (3), Endy (3), and Elowitz (2).

#### Genome engineering

The oldest key reference for this approach dates back to 2000, which corresponds to the period of completion of major genome sequencing projects, and the shift from genomics to post-genomics. Articles in this cluster tackled two main challenges: 1) Synthesis of large nucleic acid sequences [27]; [28–30] with terms in the lexical cluster such as “DNA assembly”, “DNA fragment” or “gene assembly” (Fig 4). 2) Research on a minimal genome [31, 32]correlated to the cluster “Minimal genome” (Fig 4) and terms such as “essential gene”, “synthetic genome” or “minimal genome”. The key steps from the John Craig Venter Institute (JCVI) consisted of synthetizing the entire genome of mycoplasma genitalium assembly of the genome (“Gibson assembly” which becomes one of the main techniques of the field, [28], genome transplantation [33] and the last step with the creation of the first organism with a synthetic genome [34]. Although an international consortium (http://syntheticyeast.org/) has now begun to work on the synthesis of an engineered yeast genome, in our corpus this approach was mainly lead by JCVI.

#### Protocell creation

The third approach, protocell creation, gathers papers aimed at recreating minimal biological systems from scratch by building protocells [35], [36]. The question of the definition of life and its original mechanisms are central as shown in the lexical cluster “Artificial cells” (Fig 4) with terms like “synthetic cells”, “minimal cells” and “origin of life”. The visibility of this approach is decreasing since the last major reference cited was published in 2006. Current principal investigators for this approach claiming a synthetic biology approach are mainly European, centered around the work of Luisi in Rome.

#### Metabolic engineering

The metabolic engineering (cluster 4 Fig 3) approach gathers papers dealing mainly with the design and introduction of new metabolic pathways in bacteria to produce compounds of interest. The cluster includes central papers regarding the success story of artemisinic acid [37, 38]. Articles about the metabolic pathway optimization from synthetic promoter libraries can also be found [39]. The focus on applications is clearly expressed and mainly directed towards the production of biofuels [40, 41]. This pole is very strongly correlated (Fig 5) with the lexical cluster “metabolic engineering” and with terms such as “chemical and fuel products”, “Biosynthesis pathways” and “microbial factories” and also terms referring to the relationship with industry such as “industrial biotechnology”. Jay Keasling, currently professor of biological engineering at UC Berkeley, is a highly influential scientist in this cluster. He co-authored 7 of the 14 articles. Finally, the presence of the works of Stephanopoulos [42], [43] one of the founders of the field of metabolic engineering with the publication of the book ‘Metabolic Engineering’ in 1992, shows how synthetic biology integrates and transforms already existing practices. For a clarification of the links between metabolic engineering and synthetic biology by Stephanopoulos himself see [44].

This analysis of the structure of SynBio provides a better understanding of the constitutive heterogeneity of the field. Using a rigorous empirical analysis, we show the following:

- Confirmation of four broad categories used by the actors themselves;
- The centrality of the ‘biobrick engineering’ approach;
- Identification of five sub-clusters in the ‘biobrick engineering’ approach;
- Demonstration of the weak and diminishing role of the protocells approach in SynBio;
- The existence of a ‘genome engineering’

approach, dominated by JCVI, that is not closely related to the biobrick engineering approach;

Engineering biology is generally considered as the unifying characteristic of SynBio. However, engineering life science and life organisms is neither new (think of the use of the expression synthetic biology by Leduc in the early 20th Century, or more recently the take off of biotechnology in the 70s) nor is it unique. In the central approach (biobrick engineering) this challenge is deeply inspired by electronic or computer software engineering and uses concepts such as modularity, standardization, abstraction, Boolean logic, etc. [22, 24, 45]. In the other approaches, the model is closer to chemical engineering or to civil and mechanical engineering in its use of optimization of modeling of whole system-level stresses and traffic flow [46]. This heterogeneity is related to the fact that this field emerges within a hybrid logic that both relates to scientific content and promises of application. Promises allow resources to be mobilized and used to explore new research avenues. At the same time, they put pressure on the scientists and institutions involved to take practical applications into consideration.

### Identification and basic characterization of highly influential scientists

Who are the scientists involved in SynBio? How does this population evolve? Is it possible to identify highly influential scientists? How are they involved in the different approaches identified above? What are their roles in the structuring of the field? A large number of studies have shown that for a given scientific field, a small group of scientists play a key role in formulating core concepts and approaches as well as building the scientific community (see inter alia [17, 47]. Our research shows that it is possible to identify this core group from empirical scientometric data. The originality of our approach comes from the combination of three different indicators to identify members of the core-set: (1) centrality (a common indicator used in network analysis to measure to what extent a node is likely to be a passing point for other nodes in a network); (2) the cumulative impact of their publications in the corpus; and (3) their productivity (number of articles in the corpus). These three indicators are represented on a single graph (Fig 6): centrality on the horizontal axis, impact on the vertical axis, and productivity by the size of the circle. We identified 30 authors with the highest global score obtained by the product of authors’ centrality and impact. This product is also equivalent to computing the product of centrality, average impact per paper and number of publications. Please see SI for further technical details on the identification of the members of the core-set.

First, we observe an over-representation of US based scientists. Among the 30 members of the core-set, 25 work in US institutions. This is to be compared to the part of the US within world SynBio production (at least one US resident author contributes to 44% of the papers in the corpus). This raises a series of questions on the possible influence of this US core-set on the structuration of the field. The second main result is that members of the core-set are highly concentrated in the central approach ‘biobrick engineering’ (Cf. Table 1). Name of authors who belong to scientific program SynBERC as PI, affiliate PI or as member of the SAB are followed by a *. One also notes the absence of Kobayashi in the table due to the impossibility to associate his work with the approaches identified and a lack of biographical data. The six authors contributing to the genome engineering approach are less central and their impact is quite uneven, being very high for Venter and Huchison, and quite low for Segall-Shapiro or Zhao. The profile of this approach (low centrality, variable impact) reflects the specific scientific strategies of the John Craig Venter Institute (JCVI). Six members of the core set are closely related to the metabolic engineering approach. Their centrality and impact are relatively low (except for Keasling and Church). This may be explained by the overlap between metabolic engineering and synthetic biology. Finally, only one author of the core set is mainly involved in the protocell approach (Moya). This confirms the marginality of this approach in synthetic biology.

### Members of the core-set as boundary spanners

The emergence and stabilization of a new scientific field lies on a set of institutional devices that foster communication between members of the community and the sharing of methods, instruments, and a common research agenda. These institutional programs include: summer schools, dedicated conferences, specialized research centers, academic journals. Of the few scientific journals that are dedicated to the field, two are edited by Christopher Voigt (Editor-in-chief of ACS Synthetic Biology) and Ron Weiss (Editor of Systems and Synthetic Biology)., etc. [48]. We may expect that PIs are intensively involved in these institutions. As mentioned earlier, the emergence of SynBio is also related to positive interactions with its environment. This is crucial for interacting with diverse audiences (startups, venture capital, big pharma, regulators, media, etc.), building credibility and legitimacy, and attracting resources dedicated to the development of SynBio. In this section, we also examine the role of PIs in such interactions. Although they have been selected for their centrality in the scientific field, are they also very active as boundary spanners as suggested by studies of other technoscientific fields, e.g., biotechnology and nanotechnology [49]? In order to study the role of PIs in the building of the SynBio environment, we examine their involvement in three different sets of activities: (i) contribution to core SynBio institutions; (ii) interactions with companies that use new knowledge produced in SynBio and transformation into new products and processes; and (iii) contribution to initiatives aimed at building the SynBio governance, e.g., regulation and national or international research programs. We draw on qualitative indicators based on the biographical information of PIs. For each dimension we identify important properties such as the creation of start-ups, participation in an IGEM conference, or patenting activity for which each PI is either active or inactive. The sum of this binary information builds three different scores for each author. The resulting table provides a global positioning of members of the core set in those three non-academic social spaces.

The indicator “core institutions” includes the involvement of PI in the main stabilizing institutions of SynBio already identified by social science analysis. They are namely: iGEM competition (judge, advisor or laboratory registered by the contest11), the ERC program SynBERC (PI and affiliated PI), international conferences such as SBX.0 (organizer and member of the steering committee) and SEED, Synthetic Biology: Engineering, Evolution, Design, (organizer and member of the steering committee). The indicator “business development” was built from three kinds of information: participation in a Scientific Advisory Board (SAB), creation of a start-up, and ownership of patents as inventors. Finally, the indicator “governance” captures to what extent PIs take part in writing or organization of reports dedicated to SynBio governance. On a side note, we listed no less than 58 reports to create this indicator, which reflects how important governance issues are in SynBio. Each of the three indicators shows a strong involvement of members of the core-set in activities outside of research (see Table 1).

First, the central biobrick approach in academia is accompanied by a strong involvement of its members in institutions that are highly visible and play a key role in the field (iGEM, SynBERC, BBF and SEED conference). Indeed, 21 PIs of the 25 belonging to this approach are involved in at least one of these institutions. The most active PI in these three institutions (score of 3 or 4) belong to this approach. In comparison, members of others approaches seem to be less systematically involved in this kind of institutions. All the members of the Genome Engineering approach have a score less than or equal to one. Four of six PIs of the Metabolic Engineering approach have a score lower or equal to one. Importantly, although often considered as one of the founders of the domain, JC Venter is not involved in the construction of a SynBio community (score = 0) according to our indicators.

Second, members of the core-set are very involved in activities aimed at realizing the commercial promises of SynBio. Indeed, we find that almost all of the PIs (27 over 29) are involved with the economic sector, with a high “business development” score for two-thirds of them. Then, almost all PIs applied for a patent as an inventor (27 over 29). This is an exceptional high score, even though patenting academic research results has become routine in biotechnology [50]. Finally, members of the core set are also very active in companies: among the 29, 20 are members of at least one Scientific Advisory Board (SAB) and 17 have created a startup. This involvement is extremely high for members of the core set that have been identified according to their centrality in scientific production.

Third, members of the core-set are very active in hybrid initiatives that aim to promote the “sound” development of SynBio and to build its legitimacy. Indeed, about half of the core-set (13 PIs) have coordinated at least one report dedicated on one aspect of SynBio governance. However this involvement remains relatively specific as of the 13 active PIs, only one has participated in more than two reports (note that we used a very discriminating criterion for building our “governance” indicator since we considered only strong implications in the various reports (organization, editing, coordination)). Governance issues constitute a collective and institutionalized element of the discussion about SynBio development and is maturing alongside scientific and economic developments. The most representative example of the heterogeneous agenda alignment is certainly Drew Endy (six reports). The profile of activities of JC Venter is also worth mentioning. Although he is personally involved in only one but decisive report (the Presidential Commission for the Study of Bioethical Issues), his center, JCVI, is very active in governance issues. Indeed, staff members of this center that are trained in law and political sciences have prepared reports on the regulation of SynBio. We called these PIs boundary spanners because of their multiple involvement in different social spaces. It was shown how this group of scientists play a key role in the stabilization of the field and how social heterogeneity constitute crucial resources for emerging fields. The “business development” indicator is particularly strong compared to the other two indicators. The proximity of members of the core-set with commercial activities is almost systematic, suggesting that the relationships between academia and industry are particularly structuring. This asymmetry deserves all the more mention as social science literature and much of the media coverage focus on aspects of governance and open source and usually overlook the crucial importance of relationships between academia and industry.

## Conclusion

Our scientometric approach shows that SynBio has entered a phase of stabilization. Studying the dynamics of the population of scientists, we show how since 2010, SynBio has stabilized as an autonomous scientific field, while maintaining a high level of openness to new ideas and participants. First, we described the central role of the epistemic heterogeneity in the emergence process and we showed that it allows to combine promises and realizations to convince diverse audiences. While previous work on the sociology of science has shown that heterogeneity is routine in normal science, few studies have shown its role in the constitution of a new emergent scientific field. We showed that SynBio is composed of four distinct approaches and that biobrick engineering approach is the central track. These results were obtained through an original combination of co-citation and lexical analysis that showed a strong correlation between co-word and co-citation clusters specific to each approach. This allows us to assert that these identified approaches are not only rhetorical or strategic, but structuring for the epistemic landscape. Second, we identified a core-set of highly influential scientists. These key scientists have been identified after a systematic analysis of corpus authors done by combining three different indicators: (1) centrality; (2) cumulative impact of their publications in the corpus, and (3) productivity (number of articles in the corpus). We show that the members of the core-set are also boundary spanners. We claim that this collective boundary position is a central mechanism in the stabilization of the scientific community. Indeed, we show that, although they are identified because of their high impact and centrality in scientific production, they also play a part in roles that go beyond the academic space (core institutions, business sector and governance). This original methodology could be applied to other emerging fields like genome editing, personalized medicine, geoengineering, etc. Moreover, it opens a new point of view for understanding the development of SynBio from a sociological perspective. Our results invite social scientists working on this field to further consider its epistemic diversity and to investigate the industry-academy relationship.

## Supporting Information

S1 File Methodology. Detailed methodology for query expansion, mapping strategy, contingency matrix and core-set identification.
(PDF)
