# Supplementary material for: Mapping the Emergence of Synthetic Biology
Source: PLoS One. 2016 Sep 9;11(9):e0161522. doi: 10.1371/journal.pone.0161522 (PMC5017775; doi:10.1371/journal.pone.0161522)
Supplement: S1 File — Detailed methodology for query expansion, mapping strategy, contingency matrix and core-set identification. (PDF) [file pone.0161522.s001.pdf]

Supporting Information

Corpus expansion

The specificity score of a term is assessed as follows: for each term "T", two queries including or not "synthetic biology" (first: $TS = T$ and then *TS= "synthetic biology" and TS= T*) are executed, resulting in two corpora. The ratio between the number of publications in each corpus provides a measure of the specificity of term T regarding the core corpus. Only terms whose specificity was above 0.1 were selected. The complete list of article identifiers (ISIUT) that are used to build the corpus are available as a Harvard Dataverse dataset with the doi:10.7910/DVN/6V4FEK at the following address: http://bit.ly/2biA0wT

We then provided the following additional files: (i) the ISI/DOI numbers for the top cited articles used in the early part of the paper, (ii) the list of terms used to the semantic map, (iii) the access to an on line reader of our corpus allowing anyone to explore it: http://bit.ly/2bCaGPV

Mapping

The total number of nodes (in both lexical and co-citations networks) necessarily had to be stopped, but we claim this choice did not impact the resulting interpretation. We enumerated a total number of 73,000 different references in our corpus. Mapping such a large number of nodes or even a few thousand is intractable for practical reasons. Second, focusing on the 100 most cited references still allows us to consider more than 10 % of the total number of citation events. We then consider that this number is sufficient to provide a realistic account of the pool of the most salient knowledge that synthetic biology papers are keen to cite. In other words, we claim that the map resulting from the analysis of the co-citation network of the 100 most cited references does not qualitatively differ from the map that we may have obtained had we considered the 200 or even 1,000 most cited references, simply because the boundaries of epistemic groups are already retrieved when considering the top most highly cited references. Small variations may occur (clusters may split into sub-components, offering a finer-grained description for example) when adding new nodes, but the key point is that most of these new references will simply organize along the boundaries of the global landscape already detected through the analysis of the most widely cited references. If we take the authors' standpoint it also seems reasonable to think that a limited number of "landmarks" are actually used to mentally organize "field structure" (even though this perception may be significantly variable). The most cited publications are certainly the most probable candidates to play this role. The same reasoning also applies to the lexical maps.

Contingency Matrix

Details of the Construction of the contingency matrix The contingency matrix (Fig 5) illustrates how correlated (and conversely anti-correlated) the distribution of articles over co-citation and lexical clusters are. Each article is first assigned the lexical and co-citation clusters they are the closest to according to their vocabulary and reference list. Note that an article may be assigned to several clusters (or none at all) according to its profile. We then compute the contingency matrix enumerating for any pair (c,s) (co-citation cluster c and lexical cluster s) for the actual number of papers which were assigned to these clusters. A null model was used assuming the independence of each distribution to assess how much those measures differ from the theoretical measures. Fig 5 allows visualization of the strongest correlations and anti- correlation between categories. The matrix was constructed as follows: first each cluster is assigned a size proportional to the number of articles falling in this category (column width when considering lexical clusters, row height when considering co-citation clusters). Each cell then corresponds to a pair of clusters. The color index illustrates how intense the actual number of papers sharing those two clusters is above or beyond the theoretical number that would be expected if the distribution of articles over the two dimensions were totally independent. More precisely we compute the ratio between observed and expected values minus 1 if the measure exceeds the theoretical value and conversely the ratio between expected and observed values minus 1 in case of under-representation. In this last case, we add a negative sign to the final result.. Red cells then code for correlated clusters, while blue cells show anti-correlated clusters. The P-value associated with a Fischer test was also computed to check whether the observed versus expected amount of articles statistically differed with 95% confidence. (Anti-)correlations failing the test were marked with a cross.

Core-set

We are using Author Name field to identify actors (AU tag in the orignal data). But author names are not always consistent in Web Of Science records. First two distinct authors may have exactly the same name (homonymy cases). Second, the way first-names are abbreviated is not always consistent for the same person. We follow this 5 steps strategy to circumvent this issue:

1. We compute impact and centrality of the whole population of authors using their original author name (tag AU),

2. Top 30 scientists are selected according to the product of impact and centrality. The 30th on the list has a score of 0,00051.

3. We are first searching for authors whose name may appear under several forms in the dataset. It seems reasonable to limit our search to authors which name already scores higher than 0,00001 (95 authors) as we assume that author names can't appear in the database in more than a few different written forms. Although, it is hard to predict how centrality may be affected by the merging of two names our strategy is already quite conservative.

4. Every time we find an author name who was possibly a duplicated of one of the original authors among the top 95 authors, we manually check whether it is the same person or not (using full author names and institutional information). This procedure ends up in 31 merging operations:; "alper, h" merged with "alpher, hs"; "arkin, a" merged with "arkin, ap"; "benner, s" merged with "benner, sa"; "chen, f" merged with "chen, ff"; "church, g" merged with "church, gm"; "collins, j" merged with "collins, jj"; "elowitz, m" merged with "elowitz, mb"; "fussenegger, m" merged with "fussenegger, mt"; "hutchison, s" merged with "hutchison, sa"; "isaacs, f" merged with "isaacs, fj"; "keasling, j" merged with "keasling, jd"; "kim, t" merged with "kim, ty"; "kitney, ri" merged with "kitney, r"; "liao, j" merged with "liao, jc"; "lu, t" merged with "lu, tk"; "mcmillen, d" merged with "mcmillen, dr"; "medford, j" merged with "medford, ji"; "morange" merged with "morange, m"; "prather, kj" merged with "prather, klj"; "sole, r" merged with "sole, rv"; "stephanopoulos, gn" merged with "stephanopoulos, g"; "tan, c" merged with "tan, cm"; "tsimring, l" merged with "tsimring, ls"; "venter, c" merged with "venter, jc"; "voigt, c" merged with "voigt, ca"; "woolfson, d" merged with "woolfson, dn"; "zhang, y" merged with "zhang, yhp"; "zhang, yh" merged with "zhang, yhp"; "zhao, h" merged with "zhao, hm"; "you, l" merged with "you, lc"; "weber" merged with "weber, w".

5. Overall the final collaboration network is composed of 11319 author names (after merging of 31 couples of author names). We recompute the centrality, impact and number of publications for each author and build a new list of top 30 members: "collins, jj", "keasling, jd", "church, gm", "weiss, r", "hutchison, ca", "fussenegger, mt", "elowitz, mb", "arkin, ap", "voigt, ca", "hasty, j", "benner, sa", "lu, tk", "endy, d", "silver, pa", "kobayashi, s", "zhang, yhp", "weber, w", "wang, x", "mcmillen, dr", "lim, wa", "liao, jc", "venter, jc", "you, lc", "zhao, hm", "stephanopoulos, g", "lee, j", "smolke, cd", "segall-shapiro, th", "arnold, fh", "moya, a"

6. Finally we check among this list wether some authors have homonyms resulting in the exclusion of "lee, j" who indexes 6 different people having each authored one paper (their full name being "Lee, Joungmin", "LEE, J", "Lee, Jack", "Lee, Jooyoung", "Lee, Jinhwa", "Lee, Jungseok".) As a result we replace "lee, j" with the 31st person in the list: "de lorenzo, v".
